# HIV testing, risk perception, and behaviour in the British population

**DOI:** 10.1097/QAD.0000000000001006

**Published:** 2016-03-07

**Authors:** Soazig Clifton, Anthony Nardone, Nigel Field, Catherine H. Mercer, Clare Tanton, Wendy Macdowall, Anne M. Johnson, Pam Sonnenberg

**Affiliations:** a Research Department of Infection and Population Health, University College London; b HIV and STI Department, Public Health England; c Department of Social and Environmental Health Research, London School of Hygiene and Tropical Medicine, London, UK.

**Keywords:** delayed diagnosis, Great Britain, HIV infections, risk taking, sampling studies, sexual behaviour

## Abstract

**Objective:**

To examine the relationship between HIV risk behaviour, risk perception and testing in Britain.

**Design:**

A probability sample survey of the British population.

**Methods:**

We analyzed data on sexual behaviour, self-perceived HIV risk and HIV testing (excluding testing because of blood donation) from 13 751 sexually experienced men and women aged 16–74, interviewed between 2010 and 2012 using computer-assisted face-to-face and self-interviewing.

**Results:**

Altogether, 3.5% of men and 5.4% of women reported having an HIV test in the past year. Higher perceived risk of HIV was associated with sexual risk behaviours and with HIV testing. However, the majority of those rating themselves as ‘greatly’ or ‘quite a lot’ at risk of HIV (3.4% of men, 2.5% of women) had not tested in the past year. This was also found among the groups most affected by HIV: MSM and black Africans. Within these groups, the majority reporting sexual risk behaviours did not perceive themselves as at risk and had not tested for HIV. Overall, 29.6% of men and 39.9% of women who tested for HIV in the past year could be classified as low risk across a range of measures.

**Conclusion:**

Most people who perceive themselves as at risk of HIV have not recently tested, including among MSM and black Africans. Many people tested in Britain are at low risk, reflecting current policy that aims to normalize testing. Strategies to further improve uptake of testing are needed, particularly in those at greatest risk, to further reduce undiagnosed HIV infection at late diagnoses.

## Introduction

Current estimates indicate that approximately 110 000 people are living with HIV in the United Kingdom, with an overall prevalence of 2.8 per 1000 population aged 15–59. Despite substantial increases in HIV testing in Britain since 2000, and testing being more common among those at greater risk [1], late diagnosis remains a major public health concern, with around a quarter of people living with HIV unaware of their infection [2]. Early diagnosis is critical to improving outcomes for those living with HIV, and also for the prevention of onward transmission, benefiting individuals and reducing treatment costs [3,4].

Black African men and women, and MSM, bear a disproportionate burden of HIV infection in the United Kingdom, with 56 per 1000 black Africans and 59 per 1000 MSM estimated to be HIV positive. Figures from 2009 indicate that over 90% of HIV among those identifying as black African was reported to be heterosexually acquired, and around 80% was acquired in Africa [4]. Black Africans are advised to have an HIV and sexually transmitted infection screen if having unprotected sex with new or casual partners, yet the proportion of infections diagnosed late (CD4^+^ cell count <350 cells/μl) are particularly high in this group, at 66% in men and 61% in women [2]. Although late diagnoses are less common among MSM, around half of newly diagnosed MSM in 2012 had never tested before despite recommendations to test at least annually, or more often if having unprotected sex with new or casual partners [5].

National strategies have aimed to increase the uptake of testing in traditional settings such as genitourinary medicine (GUM)/sexual health clinics and expand the range of settings in which testing is offered [6–8]. Routine testing is recommended in general practice and hospital admissions for areas where the diagnosed HIV prevalence in the local population is over two per 1000; however, evidence suggests that adherence to these guidelines is poor [9–11]. Testing is now also available outside of healthcare services via point-of-care tests in community settings and postal kits wherein self-taken samples are sent to a laboratory for testing. In April 2015, self-testing kits became legally available in Britain, allowing people to test without the involvement of healthcare professionals [12]. The decision to test for HIV is likely to be affected by a range of factors, including the accessibility of testing and healthcare, perceived norms around testing, perception of personal risk and prompts to test, such as being offered a test by a healthcare provider [13–18]. Important barriers to testing include fear of a positive test result and perceived stigma (relating to both a positive diagnosis and also the act of testing) [16–18].

Understanding testing patterns in the general population is important to guide HIV testing policies at both local and national levels. Here we investigate associations between HIV risk behaviour, HIV risk perception and HIV testing in a large probability survey, the third National Survey of Sexual Attitudes and Lifestyles (Natsal-3).

## Methods

### Participants and procedures

Natsal-3 was a stratified probability sample survey of 15 162 men and women aged 16–74 years in Britain, interviewed in 2010–2012. One person per household was selected to participate, with an overall response rate of 57.7% (of all addresses known or estimated to be eligible) and a cooperation rate of 65.8% (of all eligible addresses known to be eligible). Full details of the methods [19,20] and the demographic characteristics of participants have been reported elsewhere [21].

Interviews used a combination of face-to-face [computer-assisted personal interviewing (CAPI)] and self-completion [computer-assisted self-interviewing (CASI)] questions. ‘Question wording’ is given in Appendix 1. Those reporting any sexual experience (ever) were asked the CASI questions including whether they had ever had an HIV test; if so what the reasons for this were (more than one reason could be given), and when and where they were last tested. Two hundred and seventy-two (4.4%) men and 480 (5.5%) women answering ‘maybe/not sure’ to whether they had ever been tested for HIV, and a further 15 men and 12 women who did not answer the question were excluded from analysis. In general, these participants were younger than those who did answer, but did not differ by ethnicity or reported behaviour. A CAPI question about perceived risk of HIV given one's present sexual lifestyle was asked using a showcard listing response options so that participants just reported a letter code. Sexual behaviour questions, including questions on same-sex partners, were asked in the CASI. Data on self-defined ethnicity were collected in the CAPI, with response options listed on a showcard. Educational level was defined according to school leaving age and qualifications obtained, with those who were no longer in education, and had not obtained qualifications typically gained at age 16 (GCSE's or equivalent) coded as having no academic qualifications. Those with only foreign qualifications were excluded from analysis of education level as there was insufficient information to code the level of these qualifications.

We present data for 5710 men and 8041 women aged 16–74 with some sexual experience ever (‘sexually experienced’) who answered questions on HIV testing. As HIV testing during blood donation constitutes mandatory screening, rather than an individual's choice to be tested, these people were not classified as having had an HIV test. Those who only tested in the past year because of blood donation (4.4% of men, 4.2% of women) were older, and less likely to report sexual risk behaviours, than those who had tested for other reasons (data not shown).

### Statistical analysis

Analyses were carried out in STATA (v13), accounting for the stratification, clustering and weighting of the data [22]. Data were weighted first to account for unequal probabilities of selection to the sample, then to adjust for differential nonresponse, by comparing with the age, sex and regional distribution of the British population using the 2011 census [19]. Logistic regression was used to calculate crude and age-adjusted odds ratios for associations between ‘high HIV risk perception’ (rating oneself as ‘greatly’ or ‘quite a lot’ at risk) and a number of markers of recent sexual risk behaviour: number of sexual partners, overlapping partners (‘concurrency’), perception of partner's concurrency (all past 5 years), number of partners without a condom in the past year and not using a condom at first sex with a new partner in the past year (‘unsafe sex’; excludes those who only had oral sex partners in the past year). Proportional Venn diagrams, created using the EulerAPE tool [23], represent the extent of overlap between sexual risk behaviour (‘unsafe sex’), high HIV risk perception and HIV testing (past year) in the general population. Further analysis was carried out among men reporting at least one male partner in the past 5 years (‘MSM’) and those of black African self-defined ethnic origin reporting at least one opposite-sex partner in the past 5 years (‘black Africans’), as the two groups most affected by HIV in Britain.

### Ethical approval

Natsal-3 was approved by the Oxfordshire Research Ethics Committee A (reference: 09/H0604/27).

## Results

Five thousand, seven hundred and ten men and 8041 women aged 16–74 years reporting sexual experience, ever, answered questions on HIV testing. Of these 1.5% (*n* = 87) men and 1.4% (*n* = 114) women identified as black African ethnicity and reported at least one heterosexual partner in the past 5 years, and 2.6% (*n* = 190) men reported at least one male partner in the past 5 years (‘MSM’).

### HIV testing and risk perception

Altogether 18.1% of men and 23.2% of women reported ever having an HIV test, with 3.5 and 5.4% testing in the past year (excluding testing carried out in the context of blood donation); 3.4% of men and 2.5% of women perceived themselves to be ‘greatly’ or ‘quite a lot’ at risk of HIV given their current sexual lifestyle (‘high risk perception’). HIV testing increased with increasing risk perception; however, the majority (86%) of those with high HIV risk perception had not had an HIV test in the past year (Table 1).

**Table 1:** Proportion of sexually experienced population reporting HIV testing (ever, past 5 years, past year), by self-rated risk of HIV.

			HIV test							
	Percent of population		Ever		Past 5 years		Past year		Denominators	
	%	95% CI	%	95% CI	%	95% CI	%	95% CI	unwt	wt
All men										
Self-rated current risk of HIV	100		18.1	(17.0, 19.4)	11.3	(10.4, 12.3)	3.5	(3.0, 4.1)	5710	6860
Greatly/quite a lot at risk	3.4	(3.0, 3.9)	25.3	(19.2, 32.5)	23.5	(17.7, 30.6)	14.0	(9.5, 20.2)	236	228
Not very much	20.7	(19.6, 21.9)	23.2	(20.5, 26.1)	16.3	(14.2, 18.7)	5.7	(4.6, 7.1)	1391	1399
Not at all at risk	75.9	(74.6, 77.1)	16.5	(15.2, 17.9)	9.5	(8.5, 10.5)	2.4	(1.9, 3.0)	4051	5199
All women										
Self-rated current risk of HIV	100		23.2	(22.2, 24.3)	15.0	(14.1, 15.8)	5.4	(4.9, 5.9)	8041	6958
Greatly/quite a lot at risk	2.5	(2.1, 2.9)	35.9	(28.2, 44.3)	27.5	(21.0, 35.1)	14.0	(9.6, 19.9)	212	171
Not very much	14.3	(13.5, 15.2)	32.7	(29.8, 35.7)	23.5	(20.9, 26.2)	9.8	(8.2, 11.6)	1387	995
Not at all at risk	83.2	(82.2, 84.1)	21.2	(20.1, 22.3)	13.1	(12.2, 14.0)	4.3	(3.9, 4.9)	6398	5754

Denominator is those aged 16–74 reporting sexual experience, ever. HIV testing excludes blood donation.

### Factors associated with high HIV risk perception

High risk perception was associated with being younger (aged 16–24), of nonwhite ethnic origin, not in a steady relationship, having no academic qualifications or for men identifying as gay or bisexual. After adjusting for age, high risk perception was associated with all the markers of sexual risk examined in both men and women: greater partner numbers, concurrent partners, same-sex partners (all past 5 years), not using a condom at first sex with a new partner (‘unsafe sex’) and two or more partners without a condom (both past year). Those who thought their sexual partner(s) had had a concurrent sexual relationship (‘yes’ or ‘probably’) were more likely to have high risk perception than those who answered ‘no’ or ‘probably not’ (Table 2). After adjusting for number of partners in the past 5 years, these markers of sexual risk remained associated with high risk perception for men, with the exception of concurrent partners. For women, most of the associations were no longer seen after adjusting for number of partners, although an association with reporting condom-less sex with a new partner remained (data not shown).

**Table 2:** Factors associated with rating oneself as greatly or quite a lot at risk of HIV, by sex.

	Men							Women						
	Denominators (unwt, wt)	%	95% CI	OR	aAOR	95% CI	*P* value	Denominators (unwt, wt)	%	95% CI	OR	aAOR	95% CI	*P* value
All aged 16–74	6080, 7321	3.4	(3.0–3.9)					8648, 7495	2.5	(2.1–2.9)				
Age group (years)							0.0089							0.0015
16–24	1610, 1155	5.0	(4.0–6.3)	1.00				2002, 1120	3.6	(2.7–4.7)	1.00			
25–34	1500, 1354	3.5	(2.7–4.5)	0.69				2449, 1356	3.1	(2.4–4.0)	0.87			
35–44	795, 1407	2.8	(1.9–4.2)	0.56				1202, 1441	2.8	(2.0–4.0)	0.77			
45–54	774, 1387	3.5	(2.3–5.3)	0.69				1111, 1427	2.7	(1.8–4.1)	0.75			
55–64	754, 1179	3.7	(2.4–5.4)	0.72				1018, 1225	1.2	(0.6–2.3)	0.33			
65–74	647, 839	1.6	(0.9–3.0)	0.31				866, 926	1.0	(0.5–1.9)	0.27			
Ethnic origin							<0.0001							<0.0001
White	5409, 6425	2.7	(2.3–3.2)	1.00	1.00	–		7682, 6626	1.9	(1.6–2.3)	1.00	1.00	–	
Mixed	113, 117	2.9	(0.8–9.5)	1.1	0.99	(0.28–3.47)		190, 136	9.1	(4.4–17.9)	5.09	4.29	(1.90–9.66)	
Asian/Asian British	298, 448	10.1	(7.0–14.4)	4.1	3.91	(2.51–6.11)		380, 365	6.2	(3.6–10.3)	3.35	3.07	(1.71–5.48)	
Black/Black British: African	103, 131	12.2	(6.6–21.6)	5	4.76	(2.35–9.67)		153, 135	7.5	(4.1–13.5)	4.14	3.67	(1.87–7.18)	
Black/Black British: Other	77, 101	6.4	(2.6–14.8)	2.5	2.46	(0.97–6.19)		128, 128	6.0	(2.8–12.1)	3.23	3.13	(1.45–6.77)	
Other ethnic origin	71, 85	6.8	(2.5–17.1)	2.6	2.47	(0.88–6.95)		104, 94	6.4	(2.1–18.1)	3.47	3.19	(0.97–10.53)	
Sexual identity, grouped							<0.0001							0.265
Heterosexual	5872, 7107	3.1	(2.6–3.6)	1.00	1.00	–		8366, 7281	2.4	(2.0–2.8)	1.00	1.00	–	
Gay or bisexual	185, 186	15.3	(10.1–22.7)	5.71	5.51	(3.33–9.11)		248, 181	4.5	(2.1–9.5)	1.92	1.59	(0.70–3.60)	
Relationship status							<0.0001							<0.0001
Married/civil partnership / steady relationship	3987, 5590	2.5	(2.1–3.1)	1.00	1.00	–		5843, 5614	2.0	(1.6–2.4)	1.00	1.00	–	
Not in a steady relationship	1984, 1630	6.0	(5.0–7.3)	2.47	2.37	(1.76–3.19)		2666, 1774	4.1	(3.3–5.1)	2.14	2.03	(1.46–2.81)	
Education level							<0.0001							<0.0001
No academic qualifications	1105, 1455	4.9	(3.7–6.5)	1.00	1.00	–		1537, 1513	3.2	(2.3–4.5)	1.00	1.00	–	
Academic qualifications typically gained at age 16	1888, 2295	3.9	(3.0–5.0)	0.78	0.61	(0.40–0.92)		2821, 2484	2.8	(2.1–3.6)	0.85	0.59	(0.38–0.91)	
Studying for/attained further academic qualifications	2774, 3301	2.0	(1.6–2.6)	0.40	0.29	(0.19–0.44)		3852, 3183	1.6	(1.2–2.1)	0.49	0.28	(0.17–0.45)	
Number of partners^a^, past 5 years							<0.0001							<0.0001
0–1	3304, 4589	2.3	(1.8–2.9)	1.00	1.00	–		5478, 5393	1.7	(1.3–2.2)	1.00	1.00	–	
2–4	1666, 1673	3.5	(2.6–4.7)	1.58	1.51	(1.00–2.29)		2142, 1392	4.0	(3.1–5.1)	2.40	2.21	(1.44–3.40)	
5+	737, 630	9.3	(7.2–11.8)	4.42	4.17	(2.75–6.30)		579, 336	7.6	(5.2–11.0)	4.79	4.24	(2.42–7.44)	
Number of partners^a^ without a condom, past year^b^							<0.0001							<0.0001
0	1898, 1942	3.7	(2.8–4.8)	1.00	1.00	–		2710, 2284	1.9	(1.4–2.6)	1.00	1.00	–	
1	3322, 4521	2.1	(1.6–2.7)	0.57	0.57	(0.39–0.83)		5016, 4562	2.2	(1.7–2.8)	1.14	1.04	(0.69–1.55)	
2+	606, 534	10.3	(7.9–13.2)	2.98	2.80	(1.85–4.22)		616, 356	8.2	(5.8–11.4)	4.58	3.39	(1.98–5.79)	
No condom used at first sex with a new partner, past year^c^							<0.0001							<0.0001
No	4894, 6100	2.5	(2.1–3.0)	1.00	1.00	–		7127, 6415	1.9	(1.5–2.3)	1.00	1.00	–	
Yes	960, 912	6.9	(5.4–8.7)	2.84	2.59	(1.88–3.57)		1211, 775	7.5	(5.8–9.6)	4.26	3.73	(2.60–5.35)	
Any same-sex partners, past 5 years							<0.0001							0.044
No	5883, 7122	3.1	(2.7–3.6)	1.00	1.00	–		8287, 7243	2.4	(2.0–2.8)	1.00	1.00	–	
Yes	188, 189	14.8	(9.7–21.8)	5.37	5.18	(3.12–8.58)		350, 242	5.6	(3.0–10.4)	2.47	2.02	(1.02–4.00)	
Overlap between partners, past 5 years							<0.0001							<0.0001
No	4836, 5992	2.8	(2.3–3.3)	1.00	1.00	–		7467, 6654	2.1	(1.8–2.6)	1.00	1.00	–	
Yes	1054, 1062	6.0	(4.8–7.5)	2.26	2.09	(1.53–2.84)		896, 563	6.5	(4.7–8.7)	2.10	1.86	(1.32–2.64)	
Partner was concurrent, past 5 years							<0.0001							0.0005
No/probably not	3610, 4527	2.3	(1.8–2.8)	1.00	1.00	–		5328, 4860	1.8	(1.4–2.4)	1.00	1.00	–	
Yes/probably	1888, 2050	5.0	(4.0–6.2)	2.25	2.12	(1.53–2.95)		2581, 1942	3.8	(3.0–4.8)	2.11	1.87	(1.32–2.65)	
Attended a sexual health (GUM) clinic, past 5 years							0.0003							<0.0001
No	4636, 6004	2.8	(2.4–3.4)	1.00	1.00	–		6610, 6166	2.1	(1.7–2.5)	1.00	1.00	–	
Yes	906, 778	6.4	(4.8–8.4)	2.34	2.09	(1.41–3.10)		1403, 838	5.8	(4.5–7.5)	2.96	2.25	(1.53–3.31)	

Denominator is those aged 16–74 reporting sexual experience, ever. aAOR, age-adjusted odds ratio; unwt, unweighted; wt, weighted.

^a^Same and/or opposite-sex partners.

^b^Vaginal/anal sex partners.

^c^Excluding those with only oral sex partners in the past year.

### Overlap between sexual risk behaviour, HIV risk perception and HIV testing

**Fig. 1:**
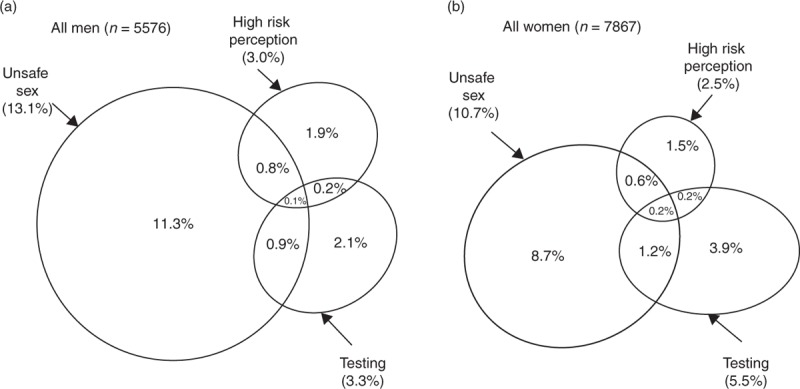
Proportional Venn diagrams showing the overlap between risk behaviour, HIV risk perception and HIV testing.

**Fig. 2:**
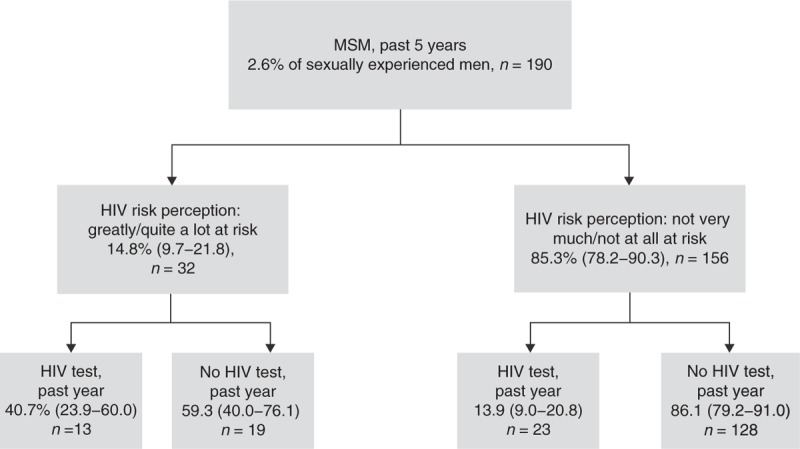
HIV risk perception and testing among MSM in the past 5 years.

**Fig. 3:**
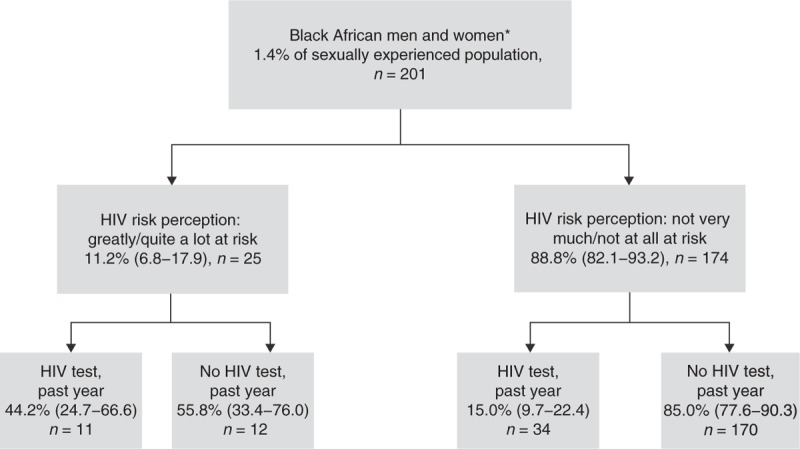
HIV risk perception and testing among black African men and women.

^∗^Reporting at least one opposite-sex partner in the past 5 years. Numbers (*n*) are unweighted and therefore do not correspond with weighted percentages presented. Owing to missing data, numbers at one level do not always add up to those at the level above.

Unsafe sex was reported by 13.1% of men and 10.7% of women, the majority of whom did not have high risk perception and had not tested for HIV in the past year (Figs. 1–3 Figs 1-3). Large proportions of those who had been tested for HIV in the past year did not report high risk perception or unsafe sex. Of those tested in the past year, 30.5% (21.2–41.7) of men and 40.7% (34.9–46.8) of women could be categorized as ‘low risk’ according to a range of measures: they reported less than two sexual partners in the past 5 years, no unprotected sex with a new partner in the past year, did not think their partner had had a concurrent partnership in the past 5 years, were not of black African ethnic origin, did not report a recent partner of black African ethnic origin, had never injected nonprescribed drugs and (for men) had not had sex with a man in the past 5 years; 73.1% (63.1–81.2) of women in this low-risk group reported ever testing because of pregnancy.

‘Unsafe sex’: Condom-less first sex with a new partner in the past year (excludes those reporting only oral sex in past year); ‘high risk perception’: rated self as ‘greatly’ or ‘quite a lot’ at risk of HIV; ‘testing’: reported HIV test, past year. Percentages shown correspond to overall percentage of the population. Denominator is those reporting at least one sexual partner, ever. Those with missing data for any variable are excluded, therefore percentages do not exactly match earlier tables.

Numbers (*n*) are unweighted and therefore do not correspond with weighted percentages presented. Owing to missing data, numbers at one level do not always add up to those at the level above.

Among MSM and black Africans, those with high HIV risk perception were more likely to report testing in the past year. However, even among these groups, large proportions [59.3% (40.0–76.1) MSM, 55.8% (33.4–76.0) black Africans] of those with high risk perception had not tested. Furthermore, the majority of those reporting recent unsafe sex (reported by *n* = 39 MSM, *n* = 51 black Africans) rated themselves as not very much/not at all at risk [83.2% (68.6–91.9) MSM and 89.3% (74.5–96.0) black Africans] and had not tested for HIV in the past year [84.8% (68.3–93.5) MSM and 78.8% (59.7–90.3) black Africans].

### Injecting drug users

Thirty-five participants reported injecting nonprescribed drugs in the past year, with 10.9% (4.8–23.2) reporting an HIV test in that timeframe; 42.8% (26.2–63.1) of these participants reported unsafe sex in the past year, which compares with 11.9% (11.3–12.5) of the general population; 8.5% (3.0–22.3) rated themselves as greatly/quite a lot at risk given their present sexual lifestyle.

### Reasons for and location of HIV testing

Of those reporting ever having an HIV test, the most commonly cited reasons were that it was part of a sexual health checkup (41.1% of men, 32.7% of women), general health checkup (26.9% of men, 10.7% of women) and, for women, in the context of a pregnancy check (47.8%). Altogether 4.8% of men and 2.6% of women said they had ever tested because they had been advised to do so by a doctor. Testing in the past year was most commonly at a sexual health (GUM) clinic (52.7% of men, 36.7% of women), GP surgery (22.0% of men, 26.3% of women) or antenatal clinic (24.1% of women) (Supplementary Tables 1 and 2). Around half of those reporting testing in the past year at general practice or hospital accident and emergency department, and a third of those tested in antenatal clinics could be classified as ‘low risk’ (as defined earlier), and more than 92% in both the groups rated themselves as not very much/not at all at risk (data not shown).

## Discussion

Using data from a large general population survey, we found that despite overall increases in HIV testing in Britain over the last decade [1], only a minority of those who perceived themselves to be at high risk of HIV had tested in the past year, including among MSM and black Africans, the population groups most affected by HIV. Furthermore, large proportions of MSM and black Africans reporting sexual risk behaviours did not perceive themselves to be at risk and had not tested in the past year. Conversely, many of those who had tested for HIV in the past year were at low risk.

Our findings complement those from qualitative and convenience sample studies in Britain, which have found low risk perception to be a barrier to HIV testing among the black African and MSM populations [17,24–27]. These studies reported that a relatively good awareness of HIV did not always translate to a perception of individual risk, possibly because of lack of symptoms, assumptions about monogamy or a lack of acknowledgement of risky behaviours [17,28]. In one survey of newly diagnosed HIV-positive Africans, almost half of participants stated that the single most important factor that could have made them test earlier would have been to be told they were at risk [25], although another study among MSM found no association between risk perception and testing [26]. We did find higher testing among those with greater risk perception; however, large proportions of those rating themselves as at risk had not tested, highlighting the importance of considering other influences on testing behaviour. These have been described by others, and include accessibility of testing and healthcare, fear of HIV and stigma, perceived norms around testing, and prompts to test, such as being offered a test by a healthcare provider [14–18]. We found that although general practice was a commonly reported setting for testing, only 4.8% of men and 2.6% of women reported testing because they were advised to do so by a doctor. This is important given evidence of missed opportunities for diagnosis of HIV in general practice [29].

The strength of this study is that it uses probability sampling to obtain a representative sample of the general population in Britain. At 57.5%, the response rate was similar to other major British social surveys, and we weighted our sample by age, sex and region to minimize nonresponse bias. Self-reported data may be subject to biases such as social desirability or recall bias; however, extensive development and cognitive testing aimed to encourage accurate reporting [19,30]. Despite this, around one in 20 participants answered ‘maybe/not sure’ to whether they had had an HIV test, suggesting problems with awareness or recall of having been tested, consistent with our previous finding that HIV testing was underreported by women who attended antenatal services [1]. HIV risk is multifaceted, incorporating individual behaviour and HIV prevalence in the sexual network, and it is not possible to completely capture an individual's true risk in a survey questionnaire. Instead, we used data on markers of sexual risk behaviour (e.g. condom-less sex with one or more new partners) and groups with known higher prevalence (MSM and black Africans) to approximate risk. This approach is likely to lead to some misclassification, but does give insight into the discrepancy between risk behaviour, risk perception and HIV testing, particularly among those in groups most affected by HIV. The small numbers of interviewees from these groups limited the precision of estimates and prohibited analysis of subgroups. This also precluded separate analysis of black African MSM, a group in whom little research has been carried out. Finally, we did not have data on all participants’ HIV status as only a subset were tested, therefore we were unable to take individuals’ HIV status into account; however, this is unlikely to be a major limitation given the low population prevalence of HIV (<0.2%) [1].

We have reported subanalyses of those identifying their ethnicity as black African, based on the UK Office for National Statistics harmonized ethnicity question, as this is most commonly used in HIV policy, statistics and reports in the United Kingdom [2,4,6,31]. However, we recognize that ethnicity is a subjective and socially constructed concept, and this category encompasses a diverse population in terms of, for example, country of birth, culture, religion and socioeconomic status [32]. Similarly, the group categorized as MSM is also heterogeneous, and, for example, does not only include men who self-identify as gay. Differences in HIV risk, knowledge and attitudes exist within these broad groups, which need to be taken into account in the design of appropriately tailored interventions [31].

The low level of risk perception found in the general population, including among those reporting sexual risk behaviours, is likely to be appropriate given the low population prevalence of HIV. More concerning were the low levels of risk perception among MSM and black Africans reporting risk behaviours, which present a major public health issue given high estimated levels of undiagnosed HIV in these populations. Given that a high proportion of people who saw themselves as at risk of HIV had not tested ever, or not tested recently, interventions need to consider other factors which have previously been found to influence testing, as described earlier. A combination of these factors have been incorporated into health promotion campaigns targeted at MSM and black Africans in Britain such as ‘HIV it starts with me’ and ‘National HIV Testing Week’, the latter of which was linked with a short-term increase in testing at GUM services among the targeted groups [33,34].

Our finding that a relatively large proportion of those tested in the past year were at low risk of HIV reflects current policies of routine testing in antenatal services, and in general practice and hospital admissions in areas with a high population prevalence (more than two diagnoses per 1000). This opt-out model aims to normalize and destigmatize HIV testing, and in doing so to increase testing in the higher-risk individuals alongside the general population [38,39]. Therefore, the finding that low-risk people are being tested does not necessarily imply mistargeted resources, although it does have implications for the cost-effectiveness of testing policy, which needs continued monitoring.

There is evidence that HIV testing is becoming more normalized, with large increases in the proportion tested at GUM services [1,2] and in community surveys, especially of MSM [35,36]. Recent innovations in testing technology, such as the availability of self-sampling and self-testing, may also help to break down barriers to testing, such as stigma, inconvenience and concern about confidentiality, reaching new populations and encouraging more regular testing among those who already test. These opportunities raise challenges which need to be managed, including addressing the possibility for user error and ensuring linkage into care and appropriate support for those who test positive [37]. People using home testing kits will also be missed from routine HIV testing data, and thus continued monitoring of testing via population surveys will be important.

Increasing uptake of HIV testing in Britain is essential to improve outcomes for those with HIV and prevent onward transmission. This article provides an understanding of testing in the context of sexual behaviour and risk perception, to guide future testing policy and interventions. Innovative strategies to improve uptake of testing are needed in those at greatest risk to further reduce undiagnosed HIV infection and late diagnoses.

## Acknowledgements

The authors thank the study participants; the team of interviewers from NatCen Social Research, operations and computing staff from NatCen Social Research; Ibi Fakoya for her valuable comments on the draft manuscript and the study funders. Natsal-3 is a collaboration between University College London, the London School of Hygiene and Tropical Medicine, NatCen Social Research, Public Health England (formerly the Health Protection Agency), and the University of Manchester.

Funding: Natsal-3 was supported by grants from the Medical Research Council (G0701757), and the Wellcome Trust (084840), with contributions from the Economic and Social Research Council and Department of Health. This report is independent research supported by the National Institute for Health Research (NIHR Research Methods Programme, Fellowships and Internships, NIHR-RMFI-2014-05-28).

This article was conceived by S.C., P.S., A.N., C.H.M., N.F., C.T. and A.M.J. S.C. wrote the first draft of the article, with further contributions from P.S., A.N., C.H.M., N.F., C.T., A.M.J. and W.M. S.C. carried out the statistical analysis, with support from P.S., C.H.M. and AMJ. P.S., C.H.M., A.M.J. and W.M., initial applicants for Natsal-3, wrote the study protocol and obtained funding. C.H.M., C.T., P.S., S.C., W.M., N.F. and A.M.J. designed the Natsal-3 questionnaire, applied for ethics approval and undertook piloting of the questionnaire. C.H.M., C.T. and S.C. managed the data. All authors interpreted data, reviewed successive drafts and approved the final version of the article.

The views expressed in this publication are those of the author(s) and not necessarily those of the NHS, the National Institute for Health Research or the Department of Health.

### Conflicts of interest

A.M.J. has been a Governor of the Wellcome Trust since 2011. The other authors declare no conflicts of interest.

## Supplementary Material

Supplemental Digital Content
