# Supplementary material for: HIV testing, risk perception, and behaviour in the British population
Source: AIDS. 2016 Mar 7;30(6):943–52. doi: 10.1097/QAD.0000000000001006 (PMC4794135; doi:10.1097/QAD.0000000000001006)
Supplement: Supplemental Digital Content [file aids-30-943-s001.docx]

**HIV testing, risk perception, and behaviour: findings from the third British National Survey of Sexual Attitudes and Lifestyles (Natsal-3)**

**SUPPLEMENTARY FILE**

**Contents**

**Appendix A:** Question wording

**Appendix B:** Supplementary tables

**Appendix 1: Question wording**

CAPI = Computer-Assisted Personal Interviewing (face to face, with showcards)

CASI = Computer-Assisted Self Interviewing (self-completion on laptop**)**

**HIV testing (CASI)**

[Apart from when you were donating blood,] have you ever had a test for HIV, the virus that causes AIDS?

1. Yes

2. No

3. Maybe/Not sure

*Text in square brackets only included if participant reported having donated blood.*

**Reasons for testing (CASI)**

Why were you tested?

You can type in more than one reason by pressing the spacebar in between each number.

1. I/ my partner was pregnant
2. for insurance or mortgage purposes or to travel to another country
3. as part of a sexual health check up
4. as part of a general health check up
5. I wanted to stop using condoms in a relationship
6. I was concerned about personal risks to myself or a partner
7. A doctor advised me to have an HIV test
8. or, other reason(s)

**When last tested (CASI)**

When was that test?

(the last HIV test if more than one)

1. In the last year

2. Between 1 and 2 years ago

3. Between 2 and 5 years ago

4. Longer than 5 years ago

**Where last tested (CASI)**

Where were you tested?

(the last HIV test if more than one)

Please choose one answer from this list.

1. General practice (GP) surgery

2. Sexual health clinic (GUM clinic)

3. NHS Family planning clinic / contraceptive clinic / reproductive health clinic

4. Ante-natal clinic / midwife

5. Private non-NHS clinic or doctor

6. Internet site offering postal kit

7. Youth advisory clinic (e.g. Brook clinic)

8. Termination of pregnancy (abortion) clinic

9. Hospital accident and emergency (A&E) department

10. Somewhere else

**HIV risk perception (CAPI)**

There are different opinions about how many people are at risk of becoming infected with HIV, the virus that causes AIDS, but we would like to know what **you** think about the risks to **you**, personally, with your present sexual lifestyle?

Just tell me the letter that corresponds to your answer.

(H) Greatly at risk

(B) Quite a lot

(W) Not very much

(S) Not at all at risk

**Appendix 2: Supplementary tables**

**Supplementary table 1: Reasons for HIV test (ever)*, by sex**

|  | Men | | Women | |
| --- | --- | --- | --- | --- |
|  | % | 95% CI | % | 95% CI |
| As part of a sexual health check up | 41.1% | [36.9%,45.4%] | 32.7% | [29.9%,35.6%] |
| As part of a general health check up | 26.9% | [23.3%,30.8%] | 10.7% | [9.0%,12.7%] |
| Pregnant / partner was pregnant | 4.5% | [3.2%,6.5%] | 47.8% | [44.8%,50.8%] |
| Concerned about personal risks to self or partner | 10.6% | [8.1%,13.9%] | 5.0% | [3.7%,6.5%] |
| Insurance or mortgage purposes, or travel to another country | 7.5% | [5.3%,10.5%] | 3.4% | [2.4%,4.9%] |
| Advised to have a test by a doctor | 4.8% | [3.4%,6.8%] | 2.6% | [1.8%,3.8%] |
| Wanted to stop using condoms in a relationship | 3.6% | [2.1%,5.9%] | 2.0% | [1.4%,2.9%] |
| Other reasons | 15.6% | [12.5%,19.3%] | 8.0% | [6.6%,9.8%] |
| *Denominators (unwt, wt)* | *746,* | *778* | *1587,* | *1040* |
| Denominator is all participants **aged 16-74** reporting sexual experience, ever, and reporting an HIV test, ever (excluding those who only had an HIV test due to blood donation). * Participants could give more than one reason, and were not asked to specifically think about the most recent test when reporting reasons | | | | |

**Supplementary table 2: Location of most recent HIV test among those tested in the past year, by sex**

|  | Men | | Women | |
| --- | --- | --- | --- | --- |
|  | % | 95% CI | % | 95% CI |
| **Location (last test)** |  |  |  |  |
| GUM clinic | 52.7% | [45.1%,60.1%] | 36.7% | [32.2%,41.3%] |
| GP surgery | 22.0% | [15.9%,29.5%] | 26.3% | [22.4%,30.5%] |
| Antenatal clinic | 0.2% | [0.0%,1.5%] | 24.1% | [20.2%,28.4%] |
| Private clinic or doctor | 5.6% | [3.1%,9.8%] | 1.9% | [0.8%,4.3%] |
| NHS Family Planning clinic | 5.9% | [3.3%,10.2%] | 3.8% | [2.4%,6.0%] |
| Hospital A&E | 5.0% | [2.3%,10.7%] | 2.9% | [1.6%,5.3%] |
| Termination of pregnancy clinic | 0.0% | - | 0.4% | [0.1%,1.4%] |
| Youth advisory clinic | 0.4% | [0.1%,1.6%] | 0.1% | [0.0%,0.7%] |
| Internet site offering postal kit | 0.0% | - | 0.2% | [0.1%,1.0%] |
| Somewhere else | 8.3% | [5.0%,13.5%] | 3.7% | [2.3%,5.7%] |
| *Denominators (unwt, wt)* | *252,* | *242* | *594,* | *375* |
| Denominator is those aged 16-74 reporting sexual experience, ever, and an HIV test in the past year (excluding those only tested due to blood donation). | | | | |
